# Experimental and theoretical examination and development of methods to calculate required pull force in wire drawing^☆^

**DOI:** 10.1016/j.heliyon.2024.e39867

**Published:** 2024-10-28

**Authors:** Joakim Larsson, Rachel Pettersson, Christer Korin

**Affiliations:** a Örebro University, 701 82, Örebro, Sweden; b Jernkontoret and KTH Royal Institute of Technology, 100 44, Stockholm, Sweden

**Keywords:** Wire drawing, drawing force, Steel wire, drawing die

## Abstract

Wire drawing is one of the oldest and most common cold metal forming processes. Wire is drawn through a single die or a set of conical dies to make it longer and stronger. In the drawing die there are three geometrical parameters that affect the drawing force: die diameter, die angle and bearing length. The force required to pull the wire through the drawing die has been investigated numerous times over the years and new drawing force equations have been developed. However, some of today's most widely used equations do not include the influence of the bearing length. In this paper the most common methods to calculate the drawing force are evaluated by means of wire drawing experiments and finite element studies. From the results, a novel equation for calculating the drawing force is proposed. The proposed equation is dependent on the bearing length and was compared with experiments and validated using finite element simulations with promising results.

## 1 Introduction

Wire drawing is one of the oldest and most used metal forming processes. Drawn wire is an important product that is used in components in electronics, vehicles, and other complex products on the market today. It is the starting point for many components such as springs, screws, clips, rivets, nails and many more.

During a wire drawing process, a metal wire is deformed as it passes through one or more conical tools called drawing dies. The cross-sectional area of the wire is reduced, and the length is increased as the wire deforms. In this process the mechanical properties of the wire change due to deformation hardening, resulting in lower ductility, plus increased hardness and yield strength of the wire material.

**Fig. 1:**
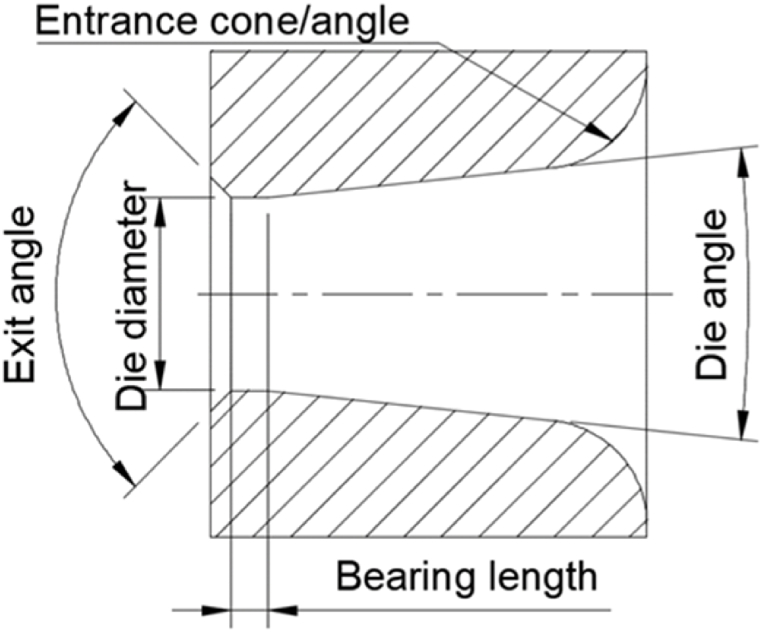
The important geometrical features of a drawing die.

The drawing die has several geometrical parameters, the three most important of which are the die diameter, the die angle and the bearing length, which are illustrated in Fig. 1. It is in the area of the die angle that the wire deforms as it is forced to follow the conical surface. When the wire reaches the bearing all deformation is done.

The force required to pull the wire through the drawing die has historically been a mystery and it is said by Persson [1] that the great Leonardo da Vinci wrote “The amount of force necessary to draw wire through a draw plate cannot be known except through experience”. During the 1900's the force requirement was investigated by several research groups, Siebel and Kobitzsch [2] derived an equation for the drawing force. Wistreich [3] performed parallel studies and came up with a similar equation. Avitzur [4,5] also derived an equation for the drawing force similar to the previous researchers. However, none of these equations include the bearing length as a parameter, implying there is no contact between the wire and the die in the bearing area. Avitzur explained that a parameter for the friction in the bearing area could be added to the equation, but then removed this parameter in a subsequent publication.

Vega et al. [6] evaluated these three equations against experiments where the die geometry was changed and came to the conclusion that both Siebel and Kobitzsch [2] and Aviztur [5] equations had close correlation to the performed experiments. Vega et al. also presents an equation which includes the bearing length. However, there is no explanation how it is derived, neither is the equation evaluated or even used.

Persson and Enghag [7] studied the influence of the length of the bearing on the drawing force. The authors concluded that a frictional part representing the frictional force in the bearing should be added to the equation for drawing force derived by Sibel and Kobitzsch [2]. Enghag [8] developed a software called PerEng for drafting and this software uses the equation proposed by Persson and Enghag for calculation of the drawing force and temperature increase depending on wire material properties and die geometry. Enghag et al. [9] also published a study in which the software was evaluated against a large number of experiments. However, they used the software as a key, iterating the friction coefficient until the same drawing force from the experiment was achieved. If all the process parameters are kept constant and only the bearing length is changed, the coefficient of friction should be similar since the surfaces and the lubricant that influence the friction are the same.

More recent Tintelecan et al. [10] and Sas-Boca et al. [11] presented studies regarding optimal die geometry and one of the parameters in these studies was the length of the bearing. Tintelecan et al. study was based on experimental work and Sas-Boca et al. study was based on modelling using finite element simulations. The study focusing on experimental work found that a particular (optimal) bearing length, neither the shortest nor the longest, resulted in the lowest drawing force. However, the results from the finite element modelling study showed that the shortest bearing length would require the least amount of drawing force. Nevertheless, the finite element study concluded that the same bearing length as in the experimental study was the optimal. The experimental setup used in Tintelecan et al.’s study is, however, not optimal for evaluating a wire drawing process. Only one meter of wire was drawn for each experiment at a speed of 0.5 m/s, meaning that each experiment lasted only 2 s. This short time is not enough for a wire drawing process to stabilize as shown by Larsson et al. [12,13]. Both Tintelecan et al. and Sas-Boca et al. concluded that the equation derived by Persson and Enghag gave accurate results regarding drawing force. However, it is unclear how the researchers came to this conclusion since the experimental results differ from the analytical results.

Suliga et al. [14] studied the effect of the bearing length on the drawing force. The authors performed both industrial experiments in a multi-draw machine and laboratory experiments. The results regarding drawing force from the industrial experiments are presented in form as percentage of motor load instead of the actual drawing force. For the laboratory experiments a drawing setup in a uniaxial tensile testing machine was used. Around 300 mm of wire was drawn at a drawing speed of 0.03 m/s. The authors state that they are aware of that this setup might not be representative for an industrial setup. They concluded that the length of the bearing does have an influence on the required drawing force and that it is beneficial to use short bearing lengths. The authors also presented a comparison between drawing force from the laboratory experiments, finite element simulations and analytical calculated values, the comparison shows that the difference between these values change as the bearing length changes.

The main focus of the present study is therefore to investigate the influence of the bearing length on the drawing force. This is done by quantitative studies in empirical experiments in a semi-industrial wire drawing process utilizing an industrial single block wire drawing machine and drawing dies with different bearing lengths.

## 2 Materials and methods

### 2.1 Experimental setup

**Fig. 2:**
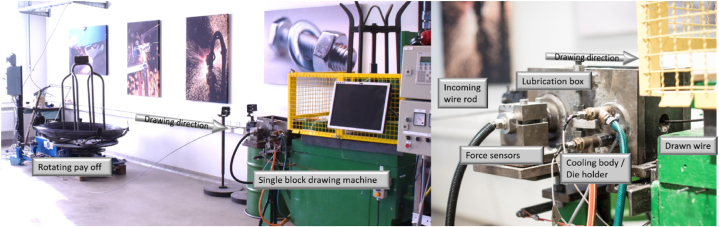
Industrial wire drawing line at Örebro university.

Wire drawing experiments were performed at Örebro university using an industrial wire drawing line setup shown in Fig. 2. The drawing line consists of a driven rotating pay off and a single block wire drawing machine. During each experiment approximately 30 m wire was drawn, which according to the acquired data was enough to generate stable steady state conditions and thus relevant measurement data.

In the present study 5 experiments were performed with constant process parameters, drawing speed 0.3 m/s, wire material from the same batch and the same type of lubricant. Only the bearing length was changed between the experiments.

#### 2.1.1 Materials

The wire material used for the experiments was a carbon steel according to EN 10270-2 VDSiCR, the material in the drawing dies was cemented carbide H10F which is a tungsten carbide with 10 % cobalt binder. Mechanical properties of the wire before and after the reduction were measured using an *Instron 4486* tensile test machine and are presented in Table 1. The chemical composition of the wire is presented in Table 2. The lubricant used for all experiments was a calcium based dry soap powder lubricant.

**Table 1:** Mechanical properties of the VDSiCr wire before and after the reduction from tensile tests of wire samples.

	Dimension (mm)	Yield Stress (MPa)	Tensile strength (MPa)
Before reduction	4.15	1050	1275
After reduction	3.8	1170	1365

**Table 2:** Chemical composition of the wire used in the experiments.

*Steel grade*	C	Si	$P_{\max}$	$S_{\max}$	Cr	Mn	Fe
VDSiCr	0.50–0.60	1.20–1.60	0.025	0.02	0.50–0.80	0.50–0.80	Balance

The drawing die geometry for all the dies was measured before the experiments using a contour measurement device (measuring the die angle and the bearing length), *Mitutoyo Contracer CV-1000* with a measurement error of less than 3 %. The die diameters were measured using an optical micrometer with an accuracy of 2 μm. Results from the measurements are presented in Table 3.

**Table 3:** Geometrical parameters for the drawing dies used in the experiments. The bearing is expressed in length (mm) and as a percentage of the die diameter.

Drawing die	Diameter (mm)	Die angle (°)	Bearing length (mm)	Bearing (%)
*1*	3.80	12.2	0.35	9.3
*2*	3.81	12.3	0.85	22.4
*3*	3.80	12.2	1.28	33.8
*4*	3.81	11.7	1.64	43.2
*5*	3.80	12.0	2.30	60.7

#### 2.1.2 Drawing force monitoring

During the experiments the drawing force was measured using two force sensors that were fitted to the drawing box. Force sensors used for this were of KIS-2 type and had a range of 0–30 kN with an error of <1 %. The lubrication box was mounted only on the force sensors, causing all forces involved in the drawing process to pass though the sensors. The signal from the sensors was sampled at 800 Hz and then processed in a *LabVIEW* [15] software, storing mean values measured signal, calculated at 1 Hz.

### 2.2 Drawing force theory

One of the most well-known ways to calculate the drawing force in a wire drawing process is to use the equation derived by Siebel and Kobitzsch [2]. The equation for the total drawing force (*F*) is divided into three parts: homogenous deformation ($F_{h}$), inhomogeneous deformation ($F_{ih}$) and friction during deformation ($F_{f}$):

(1) $$F_{h}=A_{1}R_{em}\;\ln\frac{A_{0}}{A_{1}}$$

(2) $$F_{ih}=A_{1}R_{em}\frac{2\alpha }{3}$$

(3) $$F_{f}=A_{1}R_{em}\frac{\mu }{\alpha }\ln\frac{A_{0}}{A_{1}}\;\text{.}$$

By adding Equations (1), (2), (3) the total drawing force (*F*) can be obtained

(4) $$F=A_{1}R_{em}\left(\ln\frac{A_{0}}{A_{1}}+\frac{2\alpha }{3}+\frac{\mu }{\alpha }\ln\frac{A_{0}}{A_{1}}\right)\text{,}$$

where $A_{0}$ and $A_{1}$ are the cross-sectional area of the wire before and after the reduction, $R_{em}$ is the mean yield stress of the wire material before and after the draw, 2α is the die angle and *μ* is the coefficient of friction.

As stated in the introduction and as can be seen in Equation (4) there is no parameter for the length of the bearing. The equation only adds the forces that occur in the deformation zone (the conical part). This is based on the assumption that there is no contact between the wire and the die in the calibration part (bearing zone) of the die, which is not true. After the drawing force equations were derived, finite element studies have shown that there is some contact between the wire and the die in the bearing, how much is still however unclear. Överstam [16] looked at the influence of bearing geometry on residual stresses in drawn wire. In the study both finite element modelling and wire drawing experiments were included. From the results it can be seen that when there is no contact in the bearing in the simulation, the residual stress in the wire differs the most from the experimental results, which indicates that there are some contact in the bearing between the wire and the die. This contact creates a frictional force acting opposite to the drawing direction. Persson and Enghag [7] further developed Equation (4) and added a part, Equation (5), for the frictional force in the bearing,

(5) $$F_{fb}=\pi d_{1}BR_{e1}\mu \text{,}$$

where $d_{1}$ is the diameter of the reduced wire, B is the bearing length in millimetre and $R_{e1}$ is the yield stress of the reduced wire. Adding this to Equation (4) results in

(6) $$F=A_{1}R_{em}\left(\ln\frac{A_{0}}{A_{1}}+\frac{2\alpha }{3}+\frac{\mu }{\alpha }\ln\frac{A_{0}}{A_{1}}\right)+\pi d_{1}BR_{e1}\mu \;\text{.}$$

As stated in the introduction, Avitzur [4] also had an idea how to include the bearing length in the calculation of the drawing force but excluded the term in a later publication [5]. Avitzur had a similar approach to calculate the drawing force as Siebel and Kobitzsch where the equation was built on three components which together contribute to the total drawing force, Internal – power term (*W*_*i*_), Shear – loss term (*W*_*s*_) and Friction – loss term (*W*_*f*_).

(7) $$W_{i}=2\;f\left(\alpha \right)\ln\frac{R_{0}}{R_{f}}\text{,}$$

where $R_{0}$ and $R_{f}$ are the radius of the drawn wire before and after the reduction and

(8) $$f\left(\alpha \right)=\frac{1}{sin^{2}\alpha }\left(1-\cos\;\alpha \sqrt{1-\frac{11}{12}sin^{2}\alpha }+\frac{1}{\sqrt{11*12}}\ln\frac{1+\sqrt{\frac{11}{12}}}{\sqrt{\frac{11}{12}}\cos\;\alpha +\sqrt{1-\frac{11}{12}sin^{2}\alpha }}\;\right)\text{.}$$

Further,

(9) $$W_{s}=\frac{2}{\sqrt{3}}\left(\frac{\alpha }{sin^{2}\alpha }-\cot\;\alpha \right)$$

and

(10) $$W_{f}=\frac{2}{\sqrt{3}}\;\mu \left(\cot\;\alpha \;\ln\frac{R_{0}}{R_{f}}+\frac{B}{R_{f}}\right)\;\text{.}$$

By combining Equations (7), (9), (10) an equation for the drawing force including the bearing is obtained,

(11) $$F=A_{1}R_{em}\left(2\;f\left(\alpha \right)\ln\frac{R_{0}}{R_{f}}+\frac{2}{\sqrt{3}}\left(\frac{\alpha }{sin^{2}\alpha }-\cot\;\alpha \right)+\frac{2}{\sqrt{3}}\;\mu \left(\cot\;\alpha \;\ln\frac{R_{0}}{R_{f}}+\frac{B}{R_{f}}\right)\right)\;\text{.}$$

However, Avitzur [5] subsequently simplified the equation, f(α) was approximated to 1, the term for shear loss was simplified to, $\frac{4}{3\sqrt{3}}\tan\;\alpha$, and the part for the frictional loss in the bearing was removed from the term for friction loss, resulting in,

(12) $$F=A_{1}R_{em}\left(2\;\ln\frac{R_{0}}{R_{f}}+\frac{4}{3\sqrt{3}}\tan\;\alpha +\frac{2}{\sqrt{3}}\;\mu \;\cot\;\alpha \;\ln\frac{R_{0}}{R_{f}}\right)\text{.}$$

As seen in Equation (6) and Equation (11) the yield strength of the wire is used to calculate the normal force acting between the wire and the die in the bearing area, however, as the wire already is reduced the force should be lower than this. The force acting between the wire and the die in the bearing is most probably only due to the thermal expansion and the springback of the wire.

### 2.3 Coefficient of friction

For evaluating the differences between Equations (4), (6), (11), (12) using experimental data, the coefficient of friction is of interest. This parameter represents the friction coefficient between the wire and the die surface in the complete drawing die system. The contact between the wire and the die in the deformation zone creates the largest part of the frictional force and since this part is situated in front of the bearing area, the assumption is that the coefficient of friction for the complete system should not vary if the bearing length is changed. This because the change in the total drawing force is rather small due to the change in the bearing length, which means that the process will see similar temperatures and so on. Equations (4), (6), (11), (12) have been rearranged with respect to the coefficient of friction resulting in

(13) $$\mu =\alpha \frac{F-A_{1}R_{em}\left(\ln\frac{A_{0}}{A_{1}}+\frac{2\alpha }{3}\right)}{A_{1}R_{em}\;\ln\frac{A_{0}}{A_{1}}}\text{,}$$

(14) $$\mu =\frac{F-A_{1}R_{em}\left(\ln\frac{A_{0}}{A_{1}}+\frac{2\alpha }{3}\right)}{\frac{A_{1}R_{em}\;\ln\frac{A_{0}}{A_{1}}}{\alpha }+d_{1}\pi R_{e1}B}\text{,}$$

(15) $$\mu =\frac{\frac{F}{A_{1}R_{em}}-2\;f\left(\alpha \right)\;\ln\frac{R_{0}}{R_{f}\;}-\frac{2}{\sqrt{3}}\;\left(\frac{\alpha }{sin^{2}\alpha }-\cot\;\alpha \right)}{\frac{2}{\sqrt{3}}\;\left(\cot\;\alpha \;\ln\frac{R_{0}}{R_{f}}+\frac{B}{R_{f}}\right)}$$

and

(16) $$\mu =\frac{\frac{F}{A_{1}R_{em}}\;–\;2\;\ln\frac{R_{0}}{R_{f}\;}-\frac{4}{3\sqrt{3}}\;\tan\;\alpha }{\frac{2}{\sqrt{3}}\;\cot\;\alpha \;\ln\frac{R_{0}}{R_{f}}}\text{.}$$

### 2.4 Finite element simulations

Finite element (FE) simulations were used to estimate the contact pressure between the wire and the drawing die, in the development of the equation presented in this paper.

Further, FE simulations were used to evaluate the results from the different drawing force equations compared in this study. A FE model was set up using data from the experiments: wire material properties (Table 1), die geometries (Table 3), coefficients of friction (Fig. 8) and drawing force from the experiments for validation (Table 5).

**Table 4:** Material properties used in the FE simulations.

	Thermal conductivity (W/m∗K)	Specific heat (J/kg∗K)	Density (kg/m^3^)	Young's Modulus (GPa)
Wire	45	434	7850	206
Drawing die	60	132	14900	640

**Table 5:** Mean values of the drawing force measured during the experiments.

*Bearing length (%)*	9.3	22.4	33.8	43.2	60.7
*Mean drawing force from experiments (N)*	4176	4159	4260	4201	4310

**Fig. 3:**
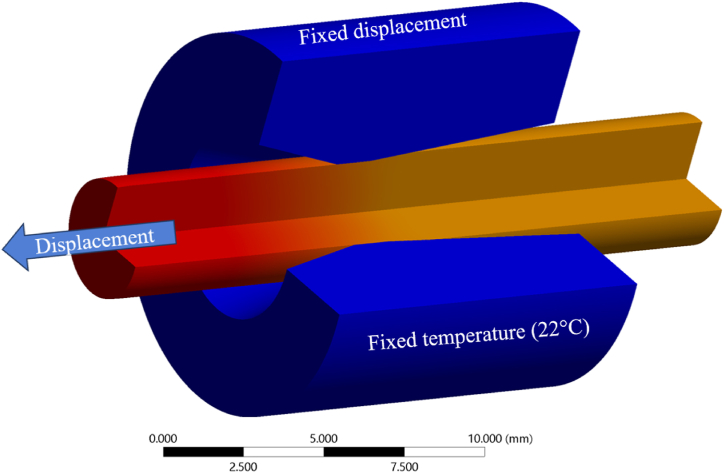
Boundary conditions used in the FE simulations.

The simulation was made using *Ansys workbench 2022* [17], where an implicit solver was used. The wire was considered to be pulled straight through the die, meaning that an axially symmetric model could be utilized for simplification. A non-linear material model with bilinear strain hardening was used for the wire. The values used in the model were collected from tensile tests performed on the wire, resulting in a hardening start value of 1050 MPa and a tangent modulus of 1.5 GPa. Other material parameters that were used in the simulations are presented in Table 4. The analysis type used was a coupled mechanical and thermal analysis, in order to capture the effect of thermal expansion of the wire. An example of the FE model with the boundary conditions is presented in Fig. 3. Only three boundary conditions were used, a displacement to pull the wire, a fixed displacement on the outer surface of the drawing die and a fixed temperature on the same surface.

The simulations were used to compare the accuracy of different drawing force equations in the following manner.

1) Calculating the coefficients of friction for the different experiments using the different drawing force equations as described in section 3.2. The resulting friction coefficients used in the FE simulations is presented in Fig. 8.
2) Using these calculated coefficients of friction in the finite element models representing each experiment. This results in five sets of coefficients of friction and five different FE models with geometries representing the five different drawing dies used in the experiments. The FE model was parameterized and the values from Table 3 was used as input to make the resulting five geometrical models. In total 25 FE simulations were performed.
3) Extracting the mean force required to pull the wire from the simulations, using the reaction force from the boundary condition moving the wire. Comparing the mean drawing force values from the force measured during the experiments with the values from the FE simulation.
4) Evaluating the difference between force in FE simulations and actual force measured in experiments, depending on which equation was used to calculate the coefficient of friction used in the FE simulations.

## 3 Results and discussion

### 3.1 Drawing force from experiments

The drawing force measured during each experiment showed slight differences between the different experiments. The mean drawing force differs by roughly 5 % (200 N) between the experiments with the highest and lowest drawing force. It was noticed that the tolerances of geometrical properties of the drawing dies (measurements presented in Table 3) have an impact on the required force, as the theory states, meaning that the order of the force levels is not in the order of the bearing length.

### 3.2 Coefficient of friction

**Fig. 4:**
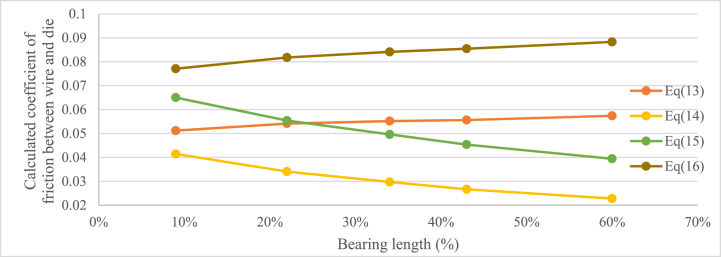
Calculated coefficient of friction using equation (13) (Siebel), equation (14) (Persson), equation (15) (Avitzur with friction in bearing) and equation (16) (Avitzur drawing force equation) depending on bearing length and method.

To evaluate the results, the friction coefficient was calculated using the mean values of the drawing force from each experiment, Equation (13), (14), (15), (16), values from Table 1, Table 3 The result, shown in Fig. 4, is that none of Equations (13), (14), (15), (16) gives a constant friction coefficient when the experiments are compared. The hypothesis is that the coefficient of friction between the die and the wire surface should be similar in the different experiments since the surface properties of the tools and the wire, the lubricant, drawing speed and reduction were the same in all the experiments.

Siebel and Kobitzsch's [2] Equation (13) and Avitzur's [5] Equation (16), that do not take the frictional force between the wire and the die in the bearing area into account, indicate that the coefficient of friction would increase with an increasing length of the bearing. This is a consequence of the evaluated drawing force being higher in the experiments with longer bearing and that there is no bearing-dependant parameter in the equation. Siebel's equation shows a change in coefficient of friction of roughly 10 % for the bearing lengths used in the experiments, this could be considered as a rather small change, so the equation should be possible to use for the tested bearing lengths with an acceptable result. For Avitzur's equation (16) the calculated coefficient of friction is higher, also the span throughout the experiments is higher, roughly 15 %. The results from Persson and Enghags's [7] Equation (13) and Avitzur's [4] Equation (15) show that the change in friction coefficient between the different experiments is greater. Both equations are seemingly overcompensating the influence of the frictional force in the bearing. The evaluated coefficient of friction differs by almost 45 % for Persson and Enghags's equation and roughly 40 % for Avitzur's equation containing a parameter for the friction in the bearing area. This large change in coefficient for the whole system due to the change of the length of the bearing indicates that the influence of this coefficient might be too large and thereby the results from the equations might be misrepresenting the friction in the process. Since Avitzur already removed the term for the friction in the bearing in the final equation for drawing force, it can be interpreted that the author came to the same conclusion regarding Equation (15).

Looking at the most recent developed equation, Persson and Enghags's Equation (6), as said in section 2.2, the authors have used the yield stress of the reduced wire as normal force for the friction force in the bearing, but since the wire is already deformed this assumption seems inaccurate. The contact pressure acting between the wire and the die in the bearing comes from the springback in the wire and the heat expansion of the wire material.

**Fig. 5:**
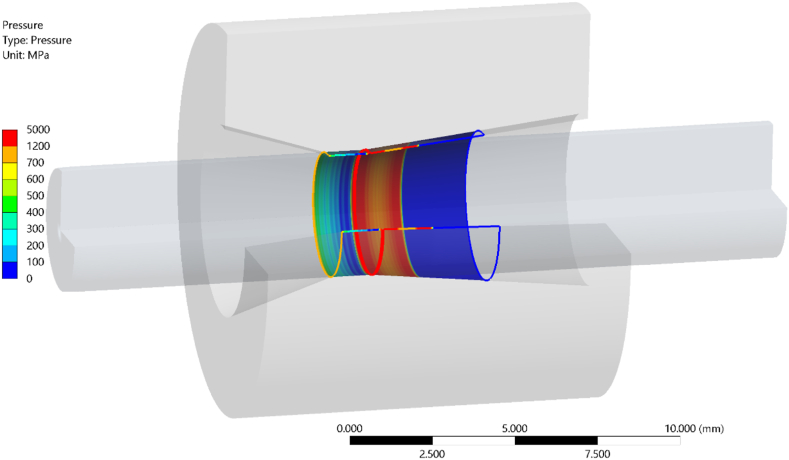
FE analysis showing the contact pressure between the wire and the drawing die.

To obtain more understanding about the contact forces that occurs in the bearing area between the wire and the die, numerical simulations were used of the type described in section 2.4. Results regarding the contact pressure between the wire and the die were analysed and for the specific case, the mean contact pressure was approximately 400 MPa, the result is shown in Fig. 5. This pressure corresponds to approximately one third of the yield stress of the reduced wire. This contact pressure will in the bearing zone act as the normal force that creates the frictional force working against the axial force (the drawing force). To verify that this assumption is valid for wire materials with different strength, additional finite element simulations were made where the yield stress for the wire material was changed (*R*_*e*_ × 1 ± 0.5), the results show that the assumption of that the contact pressure is roughly 1/3 of *R*_*e*_ is accurate for the tested yield stress span.

With the input from the performed finite element studies it is possible to add a factor of 1/3 to Equation (8), since the contact pressure is approximately 1/3 of *R*_*e*_, this results in Equation (17),

(17) $$\mu =\frac{F-A_{1}R_{em}\left(\ln\frac{A_{0}}{A_{1}}+\frac{2\alpha }{3}\right)}{\frac{A_{1}R_{em}\;\ln\frac{A_{0}}{A_{1}}}{\alpha }+d_{1}\pi \frac{1}{3}R_{e1}B}\;\text{.}$$

**Fig. 6:**
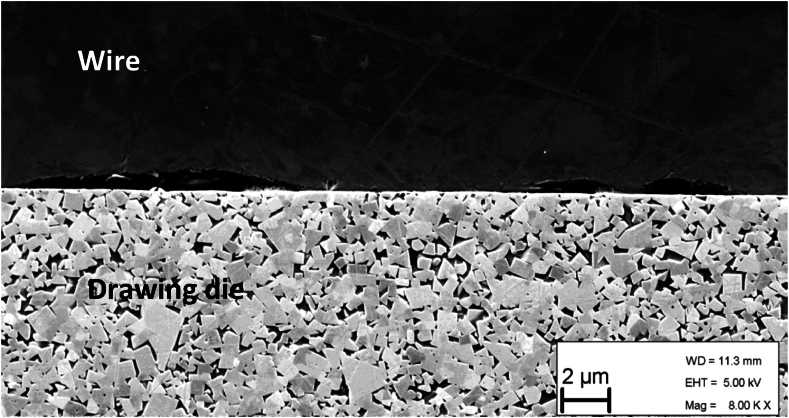
SEM study of the contact between the die and the wire in the bearing area of the drawing die, showing incomplete contact. The upper part of the picture is the wire and the lower is the drawing die.

Further, Persson and Enghags's Equation (8) assumes that there is contact between the wire and the die in the complete bearing. Fig. 6 shows an image from a scanning electron microscopy (SEM) of a drawing die that has been cut in the drawing direction with the wire still in it (Captured using a *Zeiss Ultra 55*). In the bearing area shown in the figure there is incomplete contact between the wire and the die. This is not a surprise since the literature states that there might even be no contact in the bearing area. Another factor that might affect the amount of contact is the setting of the die needed to get the correct curvature of the wire.

How much contact there is between the wire and the bearing is however unclear. To compensate for the lack of contact between the die and the wire a contact constant, C, was added to Equation (17) resulting in the following equation,

(18) $$\mu =\frac{F-A_{1}R_{em}\left(\ln\frac{A_{0}}{A_{1}}+\frac{2\alpha }{3}\right)}{\frac{A_{1}R_{em}\;\ln\frac{A_{0}}{A_{1}}}{\alpha }+d_{1}\pi \frac{1}{3}R_{e1}CB}\;\text{.}$$

**Fig. 7:**
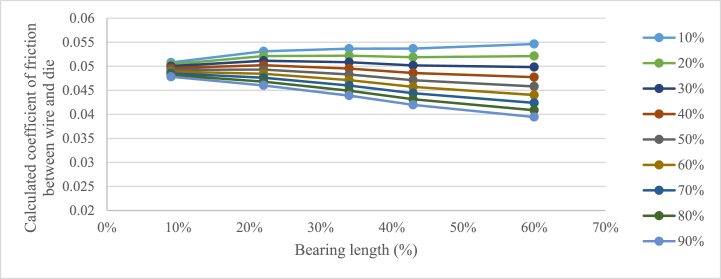
Calculated coefficient of friction for the experiments using Equation (18), varying the contact constant.

Since the hypothesis is that the friction coefficient of the system should be constant when changing the bearing length, the amount of contact in the bearing can be evaluated using Equation (18) and the results from the experiments, iterating the contact constant until a stable coefficient of friction is found. The result of this analysis is shown in Fig. 7. The contact constant was varied from 10 % contact up to 90 % contact, as can be seen in Fig. 7 the contact value that gives the least variation in friction coefficient is 30 %. Studying Fig. 6, this does not seem to be unreasonable.

**Fig. 8:**
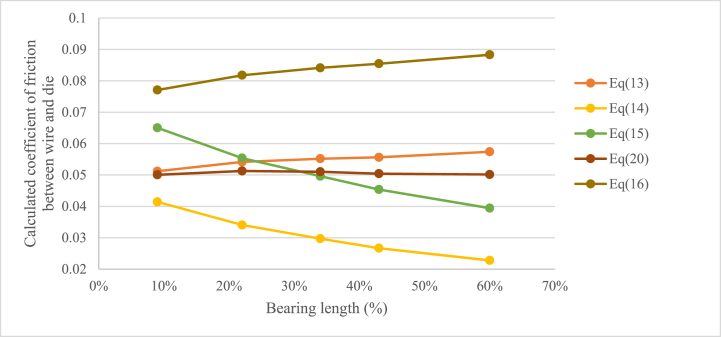
Calculated coefficient of friction using Equation (13) (Siebel and Kobitzsch), Equation (14) (Persson and Enghag), Equation (15) (Avitzur with friction in bearing), Equation (16) (Avitzur drawing force equation) and the proposed Equation (20) depending on bearing length and method.

Replacing C with the found value of 30 % contact results in:

(19) $$\mu =\frac{F-A_{1}R_{em}\left(\ln\frac{A_{0}}{A_{1}}+\frac{2\alpha }{3}\right)}{\frac{A_{1}R_{em}\;\ln\frac{A_{0}}{A_{1}}}{\alpha }+d_{1}\pi \frac{1}{3}R_{e1}0.3B}\;\text{.}$$

In order to simplify the equation and make it more user-friendly some modifications have been applied to Equation (19). To reduce the number of variables *d*_*1*_ has been replaced by expressing it as *A*_*1*_ and *R*_*e1*_ was approximated to *R*_*em*_ (which gives <0.5 % difference). Also, the input for the parameter representing the bearing length was changed. Applying these changes to Equation (19) results in the following equation:

(20) $$\mu =\frac{F-A_{1}R_{em}\left(\ln\frac{A_{0}}{A_{1}}+\frac{2\alpha }{3}\right)}{\frac{A_{1}R_{em}\;\ln\frac{A_{0}}{A_{1}}}{\alpha }+\frac{A_{1}R_{em}B\%}{250}}\text{,}$$

where *B%* is the bearing length in percent of the drawing die diameter (which is the most common way to specify the bearing length in the wire drawing industry).

When the proposed Equation (20) is used to calculate the coefficient of friction using the data from the performed wire drawing experiments, a friction coefficient that does not change with the bearing length is obtained. A comparison between Siebel and Kobitzsch's Equation (13), Persson and Enghag's Equation (14) and Avitzur's equations (15), (16) and the new proposed Equation (20) was made using the data from the experiments. The result is presented in Fig. 8 and shows that Equation (20) gives a rather constant coefficient of friction for the experiments using different bearing lengths compared to the other equations.

To achieve an equation for calculating the drawing force Equation (20) has been rearranged with respect to F, resulting in:

(21) $$F=A_{1}R_{em}\left(\ln\frac{A_{0}}{A_{1}}+\frac{2\alpha }{3}+\frac{\mu }{\alpha }\ln\frac{A_{0}}{A_{1}}+\frac{\mu B\%\;}{250}\right)\;\text{.}$$

### 3.3 Validation of results from drawing force equations using finite element simulations

To verify the performance of the different drawing force equations (4), (6)),11, 12 and 21) finite element simulations were performed as described in section 2.4. The coefficients of friction obtained using the parameters from the experiments and the five different drawing force equations (coefficients are presented in Fig. 8) was used as input to the simulations. The geometrical parameters for the drawing die and the material properties for the wire were combined with the different coefficients of friction resulting in 25 different finite element simulations (5 different geometries with 5 different coefficients of friction from the different equations).

**Fig. 9:**
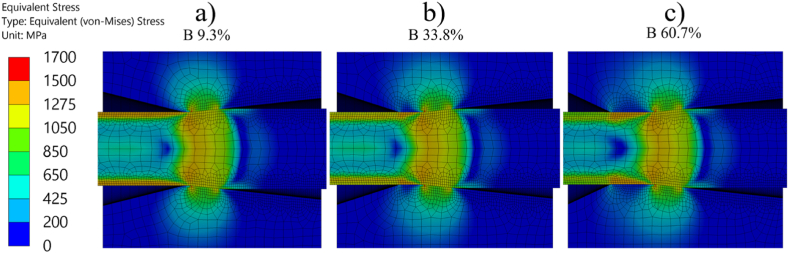
Stress distributions from three of the performed FE simulations. FE models represents the dies with a) 10 %, b) 30 % and c) 60 % bearing length. Drawing direction is to the left in the images.

Fig. 9 shows some examples of results from the FE simulations, more specific stress distributions from three simulations using three different bearing lengths and the coefficient of friction calculated using the proposed Equation (20). One interesting thing to be noted is the residual stresses in the drawn wire, the stress level in the wire surfaces decreases with an increase in bearing length.

**Fig. 10:**
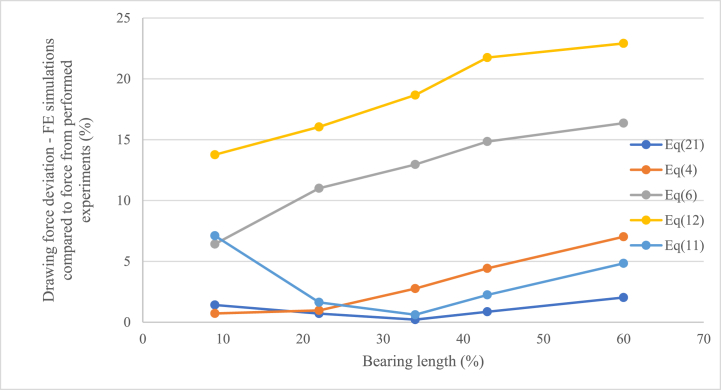
Evaluated accuracy of the compared drawing force equation (4) (Siebel), (6) (Persson), (11–12) (Avitzur) and the proposed Equation (21) comparing finite element results regarding drawing force and the measured mean drawing force from the experiments.

To compare the accuracy of the drawing force equations, the finite element simulation results regarding the drawing force (using the calculated coefficients from Equations (13), (14), (15), (16), (20) as input) was compared to the actual measured drawing force from the experiments. The result is presented in Fig. 10 as a percentage deviation of the drawing force from the finite element simulations compared to the measured drawing force.

As seen in Fig. 10, the drawing force results from the finite element simulations show a similar trend as the results regarding coefficient of friction presented in Fig. 8. As the coefficients of friction was used in the simulations to get the drawing forces, this is as expected. Using the coefficients of friction obtained by Siebel and Kobitzsch's Equation (4), Avitzur's Equation (11) and the novel Equation (21) as input gives drawing forces from the finite element simulations closest to the actual drawing force from the experiments. Around 2 % deviation for the novel equation for all the experiments and less than 5 % deviation for Siebel and Kobitzsch's Equation (4) and Avitzur's Equation (11) for bearing lengths commonly used in the steel wire drawing industry (B% ≈ 30–40 %). However, as already explained Siebel and Kobitzsch's equation does not include a parameter for the bearing length and it can be seen in the results from the finite element simulations that this results in an increasing deviation from the measured forces with an increasing bearing length (since the calculated coefficient of friction increases as seen in Fig. 8). Avitzur's equation including a parameter for the bearing length gives good results for the data obtained in the experiments, however, looking at Fig. 8, it is clear that the inaccuracy will increase with an increasing bearing length, as the coefficient of friction for the system increases with the length of the bearing. Using the coefficients of friction calculated using Avitzur's Equation (12) and Persson and Enghag's Equation (6) in the finite element simulations results in drawing forces with larger deviation from the actual drawing force for all the experiments.

## 4 Conclusion

Four of the most well-known equations for calculating the drawing force in a wire drawing process have been evaluated. Avitzur's [4,5] equations, Siebel and Kobitzsch's [2] equation and the further developed Persson and Enghag's [7] equation have been evaluated for use with drawing dies with different bearing lengths. This was done by performing, analysing and modelling of wire drawing experiments. It was found that Siebel's equation gave results close to reality when validated using finite element simulations even though no parameter for the length of the bearing is included. Also, Avitzur's [4] equation which included a parameter for the bearing length gave results close to reality when evaluated using finite element simulations. The equation derived by Persson that includes parameters for the bearing length gave results that according to the finite element simulations deviates more from the actual drawing force, the equation seemingly overcompensates the influence of the frictional force in the bearing. However, both Siebel and Kobitzsch's [2] and Avitzur's [5] equations, which gave good results when compared to the performed experiments, showed tendencies to become less accurate with an increasing bearing length.

An attempt to make an improved equation for calculating the drawing force was proposed, a modified equation (Equation (21)) was derived from Sibel and Kobitzsch's/Persson and Enghag's equations. The proposed equation takes the bearing length into account and was proposed based on experience and experimental investigations under controlled conditions.

This novel equation taking the length of the bearing into account was validated against experiments and finite element simulations. Results from the equation yield a friction coefficient between the wire and the die that does not change with the length of the bearing and resulted in the lowest deviation in drawing force when validated using finite element simulations.

## CRediT authorship contribution statement

**Joakim Larsson:** Writing – original draft, Methodology, Investigation, Conceptualization. **Rachel Pettersson:** Writing – review & editing, Supervision. **Christer Korin:** Writing – review & editing, Supervision.

## Data availability

The data used for this study will be made available on request.

## Funding

This research did not receive any specific grant from funding agencies in the public, commercial, or not-for-profit sectors.

## Declaration of competing interest

The authors declare that they have no known competing financial interests or personal relationships that could have appeared to influence the work reported in this paper.
